# Free Testosterone Drives Cancer Aggressiveness: Evidence from US Population Studies

**DOI:** 10.1371/journal.pone.0061955

**Published:** 2013-04-24

**Authors:** Shohreh Shahabi, Shiquan He, Michael Kopf, Marisa Mariani, Joann Petrini, Giovanni Scambia, Cristiano Ferlini

**Affiliations:** 1 Reproductive Tumor Biology Research, Department of Obstetrics and Gynecology, Danbury Hospital Research Institute, Danbury, Connecticut, United States of America; 2 Department of Obstetrics and Gynecology, Catholic University of the Sacred Heart, Rome, Italy; 3 Department of Oncology, Jean Paul II^nd^ Research Foundation, Campobasso, Italy; Uppsala Clinical Research Center, Sweden

## Abstract

Cancer incidence and mortality are higher in males than in females, suggesting that some gender-related factors are behind such a difference. To analyze this phenomenon the most recent Surveillance, Epidemiology and End Results (SEER) database served to access cancer survival data for the US population. Patients with gender-specific cancer and with limited information were excluded and this fact limited the sample size to 1,194,490 patients. NHANES III provided the distribution of physiologic variables in US population (n = 29,314). Cox model and Kaplan-Meier method were used to test the impact of gender on survival across age, and to calculate the gender-specific hazard ratio of dying from cancer five years following diagnosis. The distribution of the hazard ratio across age was then compared with the distribution of 65 physiological variables assessed in NHANES III. Spearman and Kolmogorov-Smirnov test assessed the homology. Cancer survival was lower in males than in females in the age range 17 to 61 years. The risk of death from cancer in males was about 30% higher than that of females of the same age. This effect was present only in sarcomas and epithelial solid tumors with distant disease and the effect was more prominent in African-Americans than Caucasians. When compared to the variables assessed in the NHANES III study, the hazard ratio almost exactly matched the distribution of free testosterone in males; none of the other analyzed variables exhibited a similar homology. Our findings suggest that male sex hormones give rise to cancer aggressiveness in patients younger than 61 years.

## Introduction

In the human species, females have longer life expectancies than males. The most recent US census data (http://www.census.gov) reports that males have a life expectancy of 75.5 years and females 80.5 years. Throughout this manuscript, we will define this phenomenon as the “gender effect”. This gender effect was masked in the past due to high rates of maternal death from childbirth [1]. The effect is now clearly visible throughout the developed world, with the only exceptions being underdeveloped countries where health systems are not capable to limit maternal deaths and the life expectancy is still that observed in developed countries one century ago [1].

Over the last two decades, the Surveillance, Epidemiology and End Results (SEER) Program of the National Cancer Institute has collected information on cancer incidence, prevalence and survival in the United States. The SEER database is freely accessible and comprises geographic areas representing 28 percent of the US population. In our opinion, this database represents a useful source to address the gender effect in cancer. An analysis using the SEER database by Cook et al. in 2009 focused on gender differences in the incidence of cancer [2]. This study clearly demonstrated that the risk of malignancy is higher in males, relative to females, for a majority of cancers at most ages. A second study by Cook et al addressed cancer mortality rate and noted a trend toward worse survival in men for a number of cancers. The authors noted that this trend tended to reflect the previously described pattern in cancer incidence [3]. One limitation of these studies is that the authors considered the gender effect as constant throughout lifetime. Indeed, at birth the differences by gender are minimal. At puberty, however, with the acquisition of sexual maturity, gender differences start to appear and ultimately peak during young adulthood. These differences begin to decrease in middle to advanced adulthood, with the decrease in gonadic sex hormone production.

The National Health and Nutrition Examination Survey (NHANES) is a survey research program conducted by the National Center for Health Statistics to assess the health and nutritional status of US population. The survey combines interviews and physical examinations, including medical, dental, and physiological measurements, as well as laboratory tests administered by medical personnel, thus providing a snapshot of the health status of the US population.

We sought to address this gap in the field by determining the relevance of the gender effect on survival analysis with respect to age as a continuous variable and possible relation to physiological variables assessed in the NHANES III population study.

## Materials and Methods

### SEER database

The April 2012 release of the 1973–2009 SEER-18 Research Data in SEER*Stat version 7.0.9 was used for this study. Information from 3,133,120 patients was initially collected and used to analyze five year cause-specific survival for all cancer sites defined in the database. Case selection was defined as actively followed cases in the research database with malignant behavior and age at diagnosis of 1 to 84 years of age. Cases with death certificate only or autopsy only, cases based on multiple primaries and cases alive with no survival time were excluded (n = 189,718). Only patients for whom information was available about race, tumor stage, tumor type, gender and age at diagnosis were included. Analysis excluded gender specific sites (ovary, endometrial, vaginal, testis and prostate cancer). Breast cancer was not included because of the disproportionate frequency by gender. Details of the ICD codes of the excluded diseases are provided in Table 1. This limited the sample size to 1,194,490 patients. The SEER cause-specific death classification was set as the definition of cause of death. The primary endpoint was cause-specific survival of each patient's originally diagnosed cancer site. Cause-specific survival was censored at the last follow-up, December 31, 2009, or five years after diagnosis, whichever came first. To analyze for gender-based survival differences, cases were stratified by males and females. Cases were further stratified by histological type and SEER Historic Stage (LRD Stage) [4].

**Table 1:** Break down of genitalia tumors excluded from the analysis.

Tissue	ICD-9 codes	Number of male patients	Number of female patients	
**Breast**	175 (males);	4,641	689,952	
	174 (females);			
**Cervix Uteri**	180;		60,076	
**Corpus Uteri**	182;		144,621	
**Other Female Genital Organs**	184; 181;		4,090	
**Other Male Genital Organs**	187;	1,150		
**Ovary**	183;		79,546	
**Penis**	187;	3,514		
**Prostate**	185;	711,145		
**Testis**	186;	34,250		
**Uterus, NOS**	179;		2,451	
**Vagina**	184;		3,006	
**Vulva**	184;		10,470	
**Total**		754,700	994,212	1,748,912

### NHANES III dataset

NHANES III is the seventh in a series of surveys that began in 1960 to examine the health of the US population. NHANES III sampled approximately 40,000 individuals from 1988 through 1994. One-hundred thirty variables related to human health were included in the analysis. All the variables were computed as provided in the dataset. The variables taken into consideration are included in the lab file available at http://www.cdc.gov/nchs/nhanes/nh3data.htm. Such variables are reported with two different scales, one in the native scale and the other one after conversion in the international system of units, thus representing 65 independent variables in two different measurement units. In particular free testosterone index was calculated according to the formula FTI (Free Testosterone Index)  =  [(TT/SHBG) * 100], as suggested by the document file attached to the dataset ftp://ftp.cdc.gov/pub/health_statistics/nchs/nhanes/nhanes3/25a/sshormon.pdf.

### Statistical analysis

Cox proportional hazards models were used to estimate the male to female hazard of cause-specific mortality, defined here as the cause of death being the specific cancer originally diagnosed and death being within five years of cancer diagnosis. A Hazard Ratio (HR) value of 1 means no difference compared to the reference, while a value lower or higher than 1 means decreased or increased risk, respectively. Multivariate analysis model included the following variables: age at diagnosis, tumor stage, cancer type (sarcoma, solid tumor or hematologic malignancy), race and gender.

Overall Survival (OS) was calculated from the date of diagnosis to the date of death or five years after diagnosis. Medians and life tables were computed using the product-limit estimate by the Kaplan-Meier method, and the Log-Rank test was employed to assess statistical significance. Analysis was performed using the same variables described above.

To assess the homology between the distribution of the HR across age and gender, the distribution of each parameter analyzed in the NHANES III dataset was computed across the available age range and the Spearman correlation test was computed to detect the presence of a statistically significant correlation. To further assess the homology between the variables an additional analysis was conducted. Two samples kolmogorov-smirnov (KS) test assessed the homology between the HR distribution and the distribution of a given variable in the NHANES III dataset. The null distribution of this statistic was calculated under the null hypothesis that the samples were drawn from the same distribution. Since the HR and the NHANES III variables have different scales, the z-score was computed for each variable according to the following equation: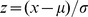, where µ and σ are mean and standard deviation of the whole population, respectively. Due to the differences of size of the two databases used for this study (SEER n = 1,194,490; NHANES III n = 29,314) we used the technique of bootstrapping (n = 10,000) to sample from the SEER database an equal number of patients capable to match for each age the size of the NHNAES III database, using the R function censboot [5]. For each bootstrap, a KS test was made and the results are expressed as % of homology, which was the % of KS tests demonstrating that the two samples were coming from the same distribution. In all cases the level of significance was set at a p value <0.05.

## Results

A multivariate Cox proportional hazard model was generated with gender, race, stage, tumor type as categorical variables and age as a continuous variable. The outcome variable was five year survival. After excluding for gender-specific cancers (Table 1), approximately 1,200,000 cases from the SEER-18 database were analyzed. All the variables included in the model were highly significant at a p<0.00001 (Table 2). Caucasians (HR 0.75 CI 0.74–0.76) had a better survival than African-Americans, while epithelial solid tumors (HR 1.8 CI 1.79–1.83) and sarcomas (HR 1.61 CI 1.57–1.66) showed a worse outcome than hematologic malignancies (reference = 1). Stage of cancer significantly affected the outcome, with patients featuring a tumor with regional (HR 0.84 CI 0.83–0.84) or local (HR 0.21 CI 0.21–0.21) involvement having a higher chance of survival as compared to patients with distant metastatic disease. Age (HR 1.03 CI 1.03–1.03) and gender (HR 1.13 CI 1.12–1.13) had a marginal but significant effect, with males exhibiting a more aggressive disease and a lower chance (∼13%) of surviving five years post-diagnosis.

**Table 2:** Multivariate Cox analysis from the SEER 18 database.

	Number of patients	Number of deaths	HR*	95% CI**	P-Value
**Gender**					<2e-16
Female	517,765	185,363	1 (Reference)		
Male	676,725	255,117	1.12	1.12–1.13	
**Age**			1.03	1.03–1.03	<2e-16
**Race**					<2e-16
African-American	127,531	58,533	1 (Reference)		
Caucasian	1,066,959	381,947	0.75	0.74–0.76	
**Tumor**					<2e-16
Hematological	98,642	35,650	1 (Reference)		
Sarcoma	21,431	5,556	1.61	1.57–1.66	
Solid	1,074,417	399,274	1.81	1.79–1.83	
**Stage**					<2e-16
Distant	417,434	224,375	1 (Reference)		
Localized	525,007	84,903	0.21	0.21–0.21	
Regional	252,049	131,202	0.84	0.83–0.84	

* HR = Hazard Ratio **CI = Confidence Interval.

**Figure 1:**
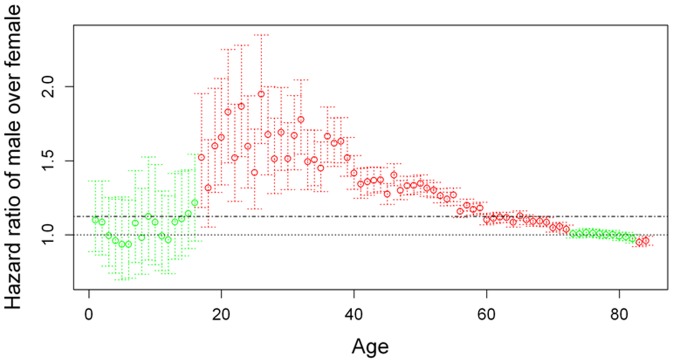
Distribution of HR (female = 1) in the age range 1–84 of patients in the SEER-18 database. Each point represents the value of HR measured with the Cox multivariate model. Bars indicate the CI interval. Green, not significant; Red, significant (P<0.05). The dotted line is the reference while the dashed indicates the HR value obtained without stratification by age (1.126). HR is not significant in the age range 1–17. In the age 18–61 it is constantly higher than 1.126 while after 62 is lower. At age 74 is not longer significant, to become again significant after 83 with a value lower than 1.

**Figure 2:**
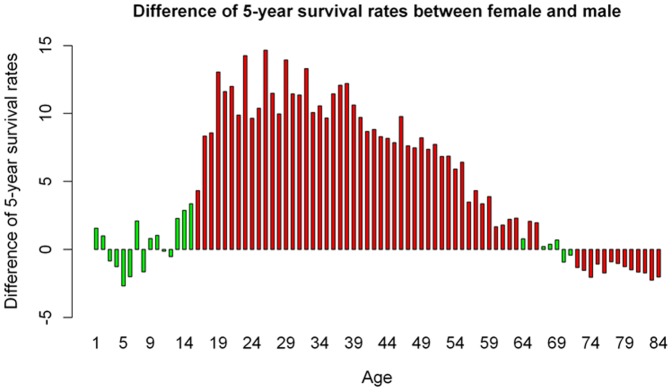
Difference in 5 years survival calculated with the Kaplan Meier method. A positive value means that females have a survival advantage as compared with males. Green, not significant; Red, significant (P<0.05). In the interval 17–63 males exhibited the worst outcome as compared with females with differences averaging more than 10% until the age of 45.

**Figure 3:**
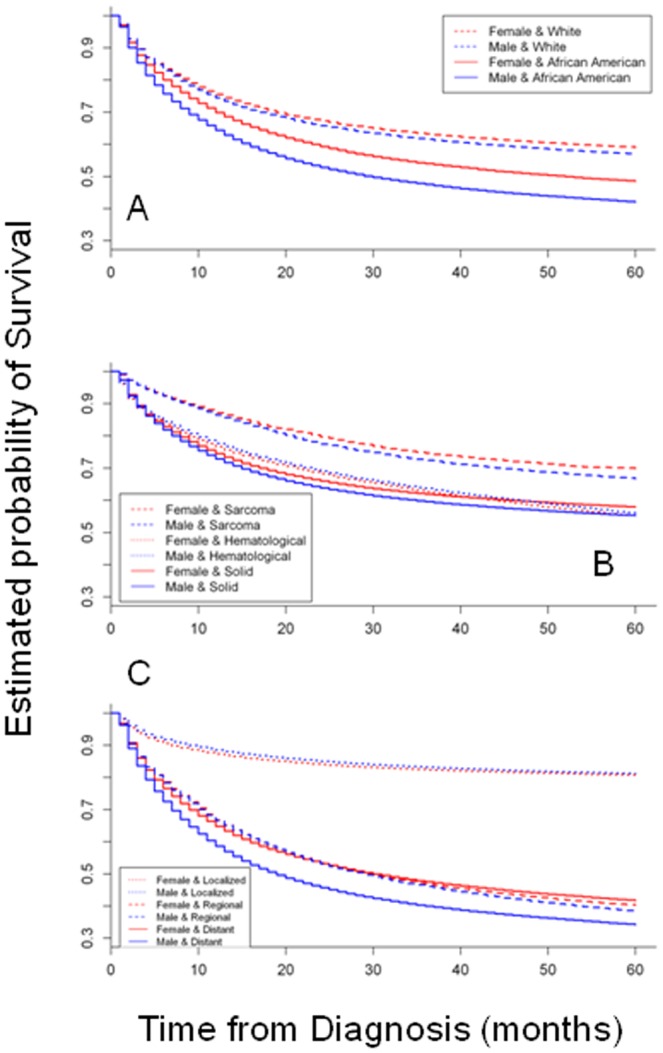
Kaplan-Meier plot according to A: Race. Blue, males; Red, females; Continuous lines, African-American; dashed lines, Caucasian; B: Type of tumor; Blue, males; Red, females; Continuous, epithelial solid tumors; dotted, hematologic malignancies; dashed, sarcomas; C: Tumor stage, Blue, males; Red, females; Continuous, distant; dotted, regional; dashed, localized disease. The major by gender differences are evident in conditions where tumors are featured by high mortality.

Thereafter, we adopted the same Cox proportional hazard model and calculated the HR over the entire age range (0 to 84 years). As depicted in Fig. 1, we used females as reference (HR = 1). No significant effects were noticed in the age range 0–17 years. From 18 to 41 years, the HR increased to average at about 1.5 and began to decrease thereafter. At the age of 61 years, the HR was below 1.13, not significant at the age of 74 years and significantly less than 1 at the age of 83 years. This led us to stratify patients in two age ranges: 17–61 years (Table 3) and 62–84 years (Table 4). We applied the same model and again, found that all variables were highly significant at p<0.00001. The gender effect was more prominent in the age range 17–61 years, with a difference of about 30% in terms of the HR compared with patients over the age of 62 years. To further investigate this phenomenon, Kaplan-Meier analysis was conducted with the same dataset. Differences in survival between females and males were computed at each age and Log-Rank test was used to assess if variation was significant at a p value <0.05 (Figure 2, Video S1). Until the age of 17 years, no significant changes were noticed. Starting from 18 years of age, an increasing and statistically significant difference was found. This effect peaked around 27 years and slightly decreased thereafter, remaining significant until the age of 63 years. After 70 years, the opposite phenomenon was noticed, with males having a slight but significant survival advantage. In terms of racial groups, the gender effect was more prominent in African-Americans than in Caucasians (Fig. 3A). In terms of tumor type, the gender effect was equally represented in sarcomas and epithelial solid tumors, but not in hematopoietic malignancies (Fig. 3B). In terms of staging, the gender effect was maximal in the most aggressive tumors with metastatic disease, slightly displayed in tumors with regional involvement and inverted in patients with localized disease, with males barely outliving females (Fig. 3C).

**Table 3:** Multivariate Cox analysis from the SEER 18 database for patients stratified for age range 17–61.

	Number of patients	Number of deaths	HR*	95% CI**	P-Value
**Age 17**–**61**					
**Gender**					<2e-16
Female	214,647	51,416	1 (Reference)		
Male	282,084	89,748	1.31	1.30–1.33	
**Age**			1.03	1.03–1.03	<2e-16
**Race**					<2e-16
African-American	61,907	25,479	1 (Reference)		
Caucasian	434,824	115,685	0.69	0.68–0.70	
**Tumor**					<2e-16
Hematological	37,012	11,299	1 (Reference)		
Sarcoma	12,274	2,765	1.87	1.79–1.95	
Solid	447,445	127,100	2.10	2.05–2.14	
**Stage**					<2e-16
Distant	149,360	72,675	1 (Reference)		
Localized	238,439	23,656	0.14	0.14–0.15	
Regional	108,932	44,833	0.68	0.67–0.69	
**Gender**					<2e-16

* HR = Hazard Ratio **CI = Confidence Interval.

**Table 4:** Multivariate Cox analysis from the SEER 18 database for patients stratified for age range 62–84.

	Number of patients	Number of deaths	HR*	95% CI**	P-Value
**Gender**					<2e-16
**Age 62**–**84**					
Female	297,522	133,296	1 (Reference)		
Male	388,217	164,464	1.04	1.03–1.04	
**Age**			1.03	1.03–1.03	<2e-16
**Race**					<2e-16
African-American	64,346	32,807	1 (Reference)		
Caucasian	621,393	264,953	0.81	0.79–0.82	
**Tumor**					<2e-16
Hematological	53,763	23,409	1 (Reference)		
Sarcoma	6,655	2,279	1.40	1.33–1.46	
Solid	625,321	272,072	1.70	1.68–1.72	
**Stage**					<2e-16
Distant	259,208	150,582	1 (Reference)		
Localized	284,555	61,033	0.26	0.25–0.26	
Regional	141,976	86,145	0.92	0.92–0.93	

* HR = Hazard Ratio **CI = Confidence Interval.

**Figure 4:**
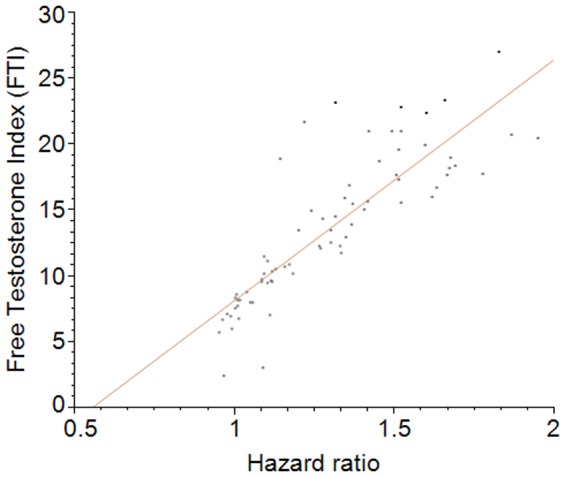
Dot Plot showing the correlation (orange line) between FTI (Y-axis) and HR (X-axis). Each data point (n = 72) is the median of the values for the range 12–84.

To identify potential biological causes of the gender effect, we analyzed a series of physiologic variables assessed in the NHANES III study population. All variables were computed across the age range for the available population sample (n = 29,314). Spearman correlation test by gender was made between the distribution of HR and each of the NHANES III variables (Table 5). The strongest correlation was noticed for Free Testosterone Index (FTI) in males with an R value of 0.9 (Fig. 4).

**Table 5:** Homology of the NHANES III parameters with the distribution of HR calculated with the Spearman correlation test.

Name*	Variable	R-Female**	P-value Female	R-Male**	P-value Male
AAP	Serum apolipoprotein AI (mg/dL)	−0.192	0.08641129	−0.281	0.01092914
AAPSI	Serum apolipoprotein AI: SI (g/L)	−0.192	0.08641129	−0.281	0.01098927
ABP	Serum apolipoprotein B (mg/dL)	−0.271	0.01447273	−0.042	0.71236907
ABPSI	Serum apolipoprotein B: SI (g/L)	−0.271	0.01427207	−0.042	0.71236907
ACP	Serum alpha carotene (ug/dL)	−0.357	0.00106457	−0.269	0.01525548
ACPSI	Serum alpha carotene: SI (umol/L)	−0.357	0.00106457	−0.249	0.02525033
AMP	Serum albumin (g/dL)	0.337	0.00356104	0.749	0.00000000
AMPSI	Serum albumin: SI (g/L)	0.339	0.00339225	0.749	0.00000000
APPSI	Serum alkaline phosphatase: SI (U/L)	−0.813	0.00000000	−0.417	0.00023949
ASPSI	Aspartate aminotransferase: SI(U/L)	−0.798	0.00000000	0.589	0.00000004
ATPSI	Alanine aminotransferase: SI (U/L)	0.099	0.40254662	0.772	0.00000000
BCP	Serum beta carotene (ug/dL)	−0.627	0.00000000	−0.814	0.00000000
BCPSI	Serum beta carotene: SI (umol/L)	−0.627	0.00000000	−0.811	0.00000000
BUP	Serum blood urea nitrogen (mg/dL)	−0.749	0.00000000	−0.735	0.00000000
BUPSI	Serum blood urea nitrogen: SI (mmol/L)	−0.750	0.00000000	−0.735	0.00000000
BXP	Serum beta cryptoxanthin (ug/dL)	−0.575	0.00000002	0.114	0.30923053
BXPSI	Serum beta cryptoxanthin: SI (umol/L)	−0.575	0.00000002	0.114	0.30923053
C1P	Serum C-peptide (pmol/mL)	−0.853	0.00000000	−0.900	0.00000000
C1PSI	Serum C-peptide: SI (nmol/L)	−0.853	0.00000000	−0.900	0.00000000
C3PSI	Serum bicarbonate: SI (mmol/L)	−0.719	0.00000000	−0.146	0.21736862
CAPSI	Serum total calcium: SI (mmol/L)	−0.360	0.00176134	0.649	0.00000000
CEP	Serum creatinine (mg/dL)	−0.735	0.00000000	−0.560	0.00000026
CEPSI	Serum creatinine: SI (umol/L)	−0.735	0.00000000	−0.564	0.00000021
CHP	Serum cholesterol (mg/dL)	−0.632	0.00000000	−0.300	0.00991549
CHPSI	Serum cholesterol: SI (mmol/L)	−0.632	0.00000000	−0.300	0.00991549
CLPSI	Serum chloride: SI (mmol/L)	0.749	0.00000000	0.305	0.00874252
CRP	Serum C-reactive protein (mg/dL)	0.120	0.28496126	−0.526	0.00000045
DWP	Platelet distribution width (%)	−0.209	0.05619882	−0.273	0.01210983
EPP	Erythrocyte protoporphyrin (ug/dL)	0.129	0.24269588	−0.821	0.00000000
EPPSI	Erythrocyte protoporphyrin: SI (umol/L)	0.129	0.24269588	−0.821	0.00000000
FBP	Plasma fibrinogen (mg/dL)	−0.785	0.00000000	−0.875	0.00000000
FBPSI	Plasma fibrinogen: SI (g/L)	−0.785	0.00000000	−0.875	0.00000000
FEP	Serum iron (ug/dL)	0.245	0.02495242	0.810	0.00000000
FEPSI	Serum iron: SI (umol/L)	0.245	0.02495242	0.811	0.00000000
FHPSI	Serum FSH: SI (IU/L)	−0.923	0.00000000	NA	NA
FOP	Serum folate (ng/mL)	−0.848	0.00000000	−0.878	0.00000000
FOPSI	Serum folate: SI (nmol/L)	−0.849	0.00000000	−0.878	0.00000000
FRP	Serum ferritin (ng/mL)	−0.309	0.00425086	0.313	0.00377445
FRPSI	Serum ferritin: SI (ug/L)	−0.309	0.00425086	0.313	0.00377445
FTI	Free Testosterone Index	NA	NA	0.896	0.00000000
G1P	Plasma glucose (mg/dL)	−0.935	0.00000000	−0.893	0.00000000
G1PSI	Plasma glucose: SI (mmol/L)	−0.935	0.00000000	−0.894	0.00000000
GBP	Serum globulin (g/dL)	0.493	0.00000959	−0.508	0.00000442
GBPSI	Serum globulin: SI (g/L)	0.493	0.00000959	−0.508	0.00000442
GGPSI	Gamma glutamyl transferase: SI(U/L)	−0.309	0.00784222	0.300	0.00982023
GHP	Glycated hemoglobin: (%)	−0.458	0.00001750	−0.328	0.00281080
GRP	Granulocyte number (Coulter)	0.330	0.00219745	−0.156	0.15681130
GRPPCNT	Granulocyte percent (Coulter)	0.099	0.36796983	−0.209	0.05677155
HDP	Serum HDL cholesterol (mg/dL)	−0.026	0.81885388	−0.028	0.80150034
HDPSI	Serum HDL cholesterol: SI (mmol/L)	−0.026	0.81647053	−0.033	0.77302280
HGP	Hemoglobin (g/dL)	−0.201	0.06652643	0.888	0.00000000
HGPSI	Hemoglobin: SI (g/L)	−0.207	0.05936647	0.887	0.00000000
HTP	Hematocrit (%)	−0.230	0.03530110	0.882	0.00000000
HTPSI	Hematocrit: SI (L/L = 1)	−0.261	0.01662213	0.882	0.00000000
I1P	Serum insulin (uU/mL)	−0.388	0.00140917	−0.631	0.00000002
I1PSI	Serum insulin: SI (pmol/L)	−0.397	0.00106616	−0.646	0.00000001
ICPSI	Serum normalized calcium: SI (mmol/L)	−0.181	0.12583385	0.592	0.00000003
LCP	Serum LDL cholesterol (mg/dL)	−0.649	0.00000000	−0.278	0.01716036
LCPSI	Serum LDL cholesterol: SI (mmol/L)	−0.648	0.00000000	−0.279	0.01664851
LDPSI	Serum lactate dehydrogenase: SI (U/L)	−0.872	0.00000000	−0.603	0.00000002
LHPSI	Serum luteinizing hormone: SI (IU/L)	−0.900	0.00000000	NA	NA
LMP	Lymphocyte number (Coulter)	0.112	0.31063595	0.111	0.31586929
LMPPCNT	Lymphocyte percent (Coulter)	−0.031	0.77844359	0.226	0.03867549
LUP	Serum lutein/zeaxanthin (ug/dL)	−0.503	0.00000166	−0.186	0.09706599
LUPSI	Serum lutein/zeaxanthin: SI (umol/L)	−0.503	0.00000166	−0.185	0.09862355
LYP	Serum lycopene (ug/dL)	0.581	0.00000001	0.693	0.00000000
LYPSI	Serum lycopene: SI (umol/L)	0.581	0.00000001	0.696	0.00000000
MCPSI	Mean cell hemoglobin: SI (pg)	−0.098	0.37316009	−0.102	0.35591627
MHP	Mean cell hemoglobin concentration	−0.025	0.81809731	0.713	0.00000000
MHPSI	Mean cell hemoglobin concentration: SI	−0.041	0.71258807	0.712	0.00000000
MOP	Mononuclear number (Coulter)	−0.459	0.00001110	−0.270	0.01296002
MOPPCNT	Mononuclear percent (Coulter)	−0.694	0.00000000	−0.303	0.00502804
MVPSI	Mean cell volume: SI (fL)	−0.162	0.14189878	−0.196	0.07470494
NAPSI	Serum sodium: SI (mmol/L)	−0.730	0.00000000	0.121	0.30861718
OSPSI	Serum osmolality: SI (mmol/Kg)	−0.774	0.00000000	−0.610	0.00000001
PBP	Lead (ug/dL)	−0.716	0.00000000	−0.366	0.00061883
PBPSI	Lead: SI (umol/L)	−0.716	0.00000000	−0.365	0.00063950
PLP	Platelet count	0.164	0.13599759	0.170	0.12316988
PLPSI	Platelet count: SI	0.164	0.13599759	0.170	0.12316988
PSP	Serum phosphorus (mg/dL)	−0.092	0.43837499	0.592	0.00000003
PSPSI	Serum phosphorus: SI (mmol/L)	−0.105	0.37698728	0.601	0.00000002
PVPSI	Mean platelet volume: SI (fL)	0.263	0.01556216	0.509	0.00000076
PXP	Serum transferrin saturation (%)	0.004	0.97279399	0.651	0.00000000
RBP	RBC folate (ng/mL)	−0.697	0.00000000	−0.829	0.00000000
RBPSI	RBC folate: SI (nmol/L)	−0.697	0.00000000	−0.830	0.00000000
RCP	Red blood cell count	−0.389	0.00025433	0.894	0.00000000
RCPSI	Red blood cell count: SI	−0.389	0.00025433	0.894	0.00000000
REP	Serum sum retinyl esters (ug/dL)	−0.478	0.00000631	0.197	0.07756829
REPSI	Serum sum retinyl esters: SI (umol/L)	−0.478	0.00000631	0.197	0.07756829
RWP	Red cell distribution width (%)	−0.213	0.05126613	−0.453	0.00001495
RWPSI	Red cell distribution width:SI(fraction)	−0.201	0.06649576	−0.466	0.00000773
SCP	Serum total calcium (mg/dL)	−0.757	0.00000000	0.498	0.00000753
SCPSI	Serum total calcium: SI (mmol/L)	−0.757	0.00000000	0.498	0.00000753
SEP	Serum selenium (ng/mL)	−0.373	0.00115565	0.050	0.67213796
SEPSI	Serum selenium: SI (nmol/L)	−0.373	0.00115565	0.049	0.67829545
SFP	Serum iron (ug/dL)	0.111	0.34785293	0.769	0.00000000
SFPSI	Serum iron: SI (umol/L)	0.120	0.31127706	0.769	0.00000000
SGP	Serum glucose (mg/dL)	−0.798	0.00000000	−0.695	0.00000000
SGPSI	Serum glucose: SI (mmol/L)	−0.798	0.00000000	−0.695	0.00000000
SKPSI	Serum potassium: SI (mmol/L)	−0.740	0.00000000	−0.764	0.00000000
TBP	Serum total bilirubin (mg/dL)	−0.160	0.17761841	0.349	0.00245347
TBPSI	Serum total bilirubin: SI (umol/L)	−0.160	0.17761841	0.349	0.00245347
TCP	Serum cholesterol (mg/dL)	−0.309	0.00506787	−0.064	0.56742948
TCPSI	Serum cholesterol: SI (mmol/L)	−0.310	0.00491528	−0.064	0.57120680
TGP	Serum triglycerides (mg/dL)	−0.432	0.00005590	−0.094	0.40159299
TGPSI	Serum triglycerides: SI (mmol/L)	−0.432	0.00005530	−0.093	0.40818301
TIP	Serum TIBC (ug/dL)	0.439	0.00002980	0.225	0.03940973
TIPSI	Serum TIBC: SI (umol/L)	0.439	0.00002920	0.225	0.03946738
TPP	Serum total protein (g/dL)	0.540	0.00000081	0.661	0.00000000
TPPSI	Serum total protein: SI (g/L)	0.540	0.00000081	0.661	0.00000000
TRP	Serum triglycerides (mg/dL)	−0.647	0.00000000	−0.356	0.00200854
TRPSI	Serum triglycerides: SI (mmol/L)	−0.648	0.00000000	−0.357	0.00191951
UAP	Serum uric acid (mg/dL)	−0.799	0.00000000	−0.118	0.31931443
UAPSI	Serum uric acid: SI (umol/L)	−0.799	0.00000000	−0.103	0.38586985
UBP	Urinary albumin (ug/mL)	−0.389	0.00040086	−0.529	0.00000054
UDP	Urinary cadmium (ng/mL)	−0.198	0.07995458	−0.264	0.01867397
UDPSI	Urinary cadmium: SI (nmol/L)	−0.197	0.08239987	−0.257	0.02218103
UIP	Urinary iodine (ug/dL)	0.090	0.43194652	−0.127	0.26442096
URP	Urinary creatinine (mg/dL)	0.665	0.00000000	0.832	0.00000000
URPSI	Urinary creatinine: SI (mmol/L)	0.666	0.00000000	0.832	0.00000000
VAP	Serum vitamin A (ug/dL)	−0.316	0.00400679	−0.125	0.26576601
VAPSI	Serum vitamin A: SI (umol/L)	−0.316	0.00402357	−0.125	0.26570892
VBP	Serum vitamin B12 (pg/mL)	−0.246	0.02668772	0.177	0.11346459
VBPSI	Serum vitamin B12: SI (pmol/L)	−0.245	0.02733610	0.177	0.11381964
VCP	Serum vitamin C (mg/dL)	−0.793	0.00000000	−0.357	0.00124030
VCPSI	Serum vitamin C: SI (mmol/L)	−0.794	0.00000000	−0.357	0.00123505
VEP	Serum vitamin E (ug/dL)	−0.390	0.00032274	−0.245	0.02737352
VEPSI	Serum vitamin E: SI (umol/L)	−0.390	0.00032274	−0.245	0.02731578
WCP	White blood cell count	0.041	0.70884419	−0.347	0.00121351
WCPSI	White blood cell count: SI	0.041	0.70884419	−0.347	0.00121351

**Figure 5:**
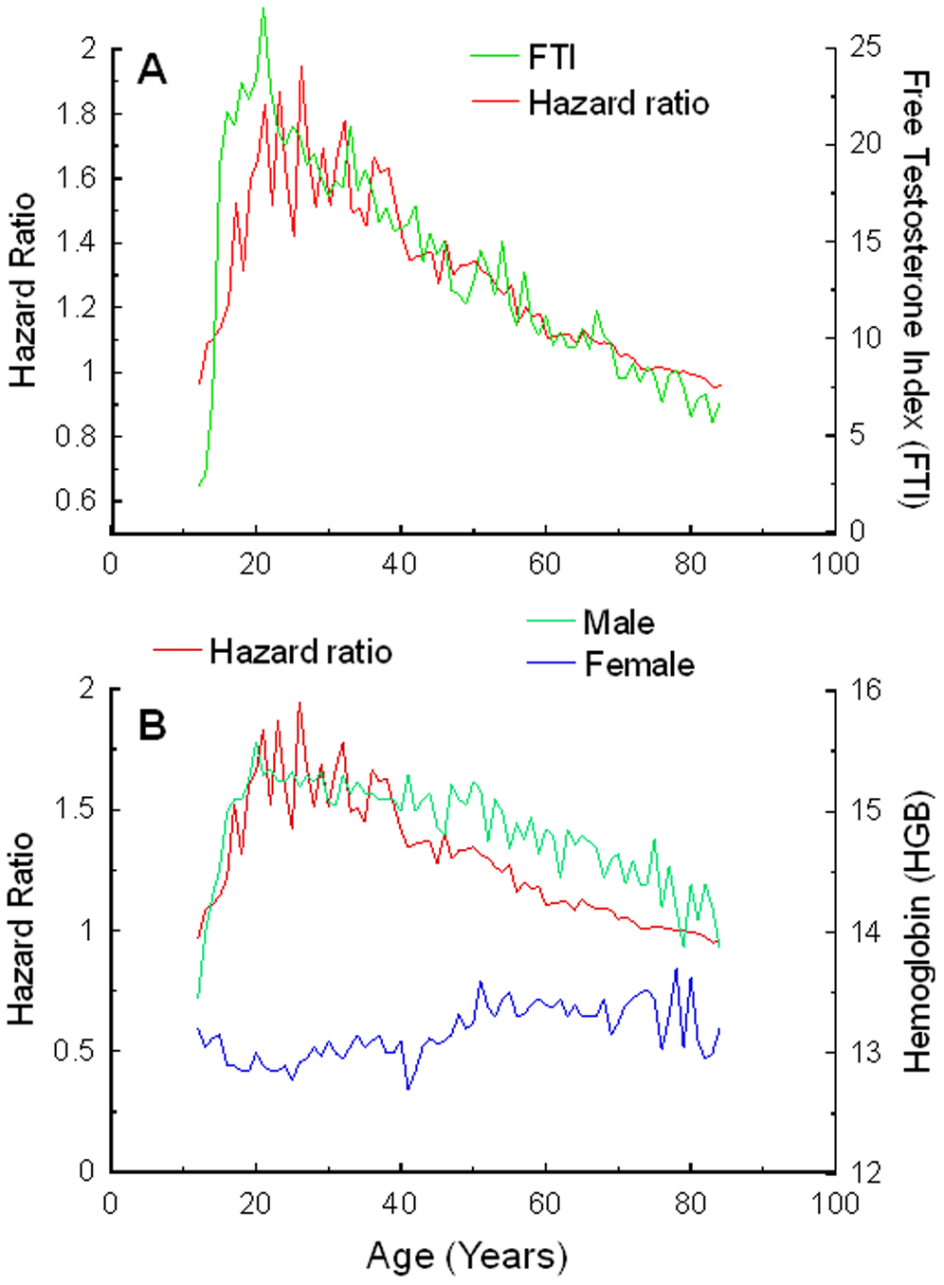
Homology between HR distribution and FTI. (A): Double Y chart reporting HR (left Y-axis; red) and FTI (right Y-axis; green) over age in years (X-axis). Homology between HR distribution and Hemoglobin (HGB) (B). Double Y chart reporting HR (left Y-axis; red) and HGB (right Y-axis; green males, blue females) over age in years (X-axis).

* Name of the variable in the attached dataset file;**calculated with Spearman Correlation test;

The homology between HR and the NHANES III was then computed for all the available variables across the available age range. KS test assessed the hypothesis that HR and a given NHANES III parameter followed in whole or in part the same distribution. This analysis was performed independently for each gender (Table 6). A striking full homology (100%) was observed for FTI in males, as the two distributions did not differ significantly across the entire age range (Fig. 5A). None of the other variables exhibited a similar degree of concordance with the HR distribution. The second strongest homology was observed for Hemoglobin in males (11.9%, Fig. 5B), where the homology was mostly confined to the age range 17–27. Noteworthy, the behavior of hemoglobin in females did not show a comparable homology with HR (Fig. 5B).

**Table 6:** Homology of the NHANES III parameters with the distribution of HR calculated with the KS method.

Number	Parameter	% Homology Female	% Homology Male
**1**	Serum apolipoprotein AI (mg/dL)	6.2%	2.5%
**2**	Serum apolipoprotein AI: SI (g/L)	6.2%	2.5%
**3**	Serum apolipoprotein B (mg/dL)	3.7%	3.7%
**4**	Serum apolipoprotein B: SI (g/L)	3.7%	3.7%
**5**	Serum alpha carotene (ug/dL)	1.2%	0.0%
**6**	Serum alpha carotene: SI (umol/L)	2.5%	0.0%
**7**	Serum albumin (g/dL)	0.0%	1.4%
**8**	Serum albumin: SI (g/L)	0.0%	1.4%
**9**	Serum alkaline phosphatase: SI (U/L)	1.4%	0.0%
**10**	Aspartate aminotransferase: SI(U/L)	1.4%	1.4%
**11**	Alanine aminotransferase: SI (U/L)	2.7%	1.4%
**12**	Serum beta carotene (ug/dL)	2.5%	1.2%
**13**	Serum beta carotene: SI (umol/L)	2.5%	1.2%
**14**	Serum blood urea nitrogen (mg/dL)	2.7%	0.0%
**15**	Serum blood urea nitrogen: SI (mmol/L)	2.7%	0.0%
**16**	Serum beta cryptoxanthin (ug/dL)	0.0%	0.0%
**17**	Serum beta cryptoxanthin: SI (umol/L)	0.0%	0.0%
**18**	Serum C-peptide (pmol/mL)	0.0%	0.0%
**19**	Serum C-peptide: SI (nmol/L)	0.0%	0.0%
**20**	Serum bicarbonate: SI (mmol/L)	0.0%	0.0%
**21**	Serum total calcium: SI (mmol/L)	0.0%	1.4%
**22**	Serum creatinine (mg/dL)	0.0%	0.0%
**23**	Serum creatinine: SI (umol/L)	0.0%	0.0%
**24**	Serum cholesterol (mg/dL)	0.0%	0.0%
**25**	Serum cholesterol: SI (mmol/L)	0.0%	0.0%
**26**	Serum chloride: SI (mmol/L)	1.4%	1.4%
**27**	Serum C-reactive protein (mg/dL)	3.7%	7.4%
**28**	Platelet distribution width (%)	2.4%	2.4%
**29**	Erythrocyte protoporphyrin (ug/dL)	4.8%	1.2%
**30**	Erythrocyte protoporphyrin: SI (umol/L)	2.4%	1.2%
**31**	Plasma fibrinogen (mg/dL)	0.0%	0.0%
**32**	Plasma fibrinogen: SI (g/L)	0.0%	0.0%
**33**	Serum iron (ug/dL)	1.2%	2.4%
**34**	Serum iron: SI (umol/L)	1.2%	2.4%
**35**	Serum FSH: SI (IU/L)	0.0%	N.A.
**36**	Serum folate (ng/mL)	2.5%	1.2%
**37**	Serum folate: SI (nmol/L)	2.5%	1.2%
**38**	Serum ferritin (ng/mL)	2.4%	0.0%
**39**	Serum ferritin: SI (ug/L)	2.4%	0.0%
**40**	Free Testosterone Index	N.A.	100.0%
**41**	Plasma glucose (mg/dL)	0.0%	0.0%
**42**	Plasma glucose: SI (mmol/L)	0.0%	0.0%
**43**	Serum globulin (g/dL)	0.0%	0.0%
**44**	Serum globulin: SI (g/L)	0.0%	0.0%
**45**	Gamma glutamyl transferase: SI(U/L)	0.0%	0.0%
**46**	Glycated hemoglobin: (%)	3.7%	6.2%
**47**	Granulocyte number (Coulter)	1.2%	2.4%
**48**	Granulocyte percent (Coulter)	3.6%	2.4%
**49**	Serum HDL cholesterol (mg/dL)	2.5%	1.2%
**50**	Serum HDL cholesterol: SI (mmol/L)	2.5%	1.2%
**51**	Hemoglobin (g/dL)	1.2%	11.9%
**52**	Hemoglobin: SI (g/L)	1.2%	10.7%
**53**	Hematocrit (%)	2.4%	10.7%
**54**	Hematocrit: SI (L/L = 1)	1.2%	10.7%
**55**	Serum insulin (uU/mL)	0.0%	0.0%
**56**	Serum insulin: SI (pmol/L)	0.0%	0.0%
**57**	Serum normalized calcium: SI (mmol/L)	0.0%	1.4%
**58**	Serum LDL cholesterol (mg/dL)	0.0%	0.0%
**59**	Serum LDL cholesterol: SI (mmol/L)	0.0%	0.0%
**60**	Serum lactate dehydrogenase: SI (U/L)	0.0%	0.0%
**61**	Serum luteinizing hormone: SI (IU/L)	0.0%	N.A.
**62**	Lymphocyte number (Coulter)	1.2%	0.0%
**63**	Lymphocyte percent (Coulter)	1.2%	0.0%
**64**	Serum lutein/zeaxanthin (ug/dL)	2.5%	3.7%
**65**	Serum lutein/zeaxanthin: SI (umol/L)	2.5%	3.7%
**66**	Serum lycopene (ug/dL)	0.0%	1.2%
**67**	Serum lycopene: SI (umol/L)	0.0%	1.2%
**68**	Mean cell hemoglobin: SI (pg)	2.4%	2.4%
**69**	Mean cell hemoglobin concentration	0.0%	2.4%
**70**	Mean cell hemoglobin concentration: SI	1.2%	1.2%
**71**	Mononuclear number (Coulter)	0.0%	1.2%
**72**	Mononuclear percent (Coulter)	0.0%	1.2%
**73**	Mean cell volume: SI (fL)	6.0%	6.0%
**74**	Serum sodium: SI (mmol/L)	1.4%	1.4%
**75**	Serum osmolality: SI (mmol/Kg)	0.0%	1.4%
**76**	Lead (ug/dL)	3.6%	6.0%
**77**	Lead: SI (umol/L)	3.6%	6.0%
**78**	Platelet count	1.2%	0.0%
**79**	Platelet count: SI	1.2%	0.0%
**80**	Serum phosphorus (mg/dL)	0.0%	1.4%
**81**	Serum phosphorus: SI (mmol/L)	0.0%	1.4%
**82**	Mean platelet volume: SI (fL)	1.2%	2.4%
**83**	Serum transferrin saturation (%)	1.2%	3.6%
**84**	RBC folate (ng/mL)	2.5%	0.0%
**85**	RBC folate: SI (nmol/L)	2.5%	0.0%
**86**	Red blood cell count	0.0%	7.1%
**87**	Red blood cell count: SI	0.0%	7.1%
**88**	Serum sum retinyl esters (ug/dL)	0.0%	0.0%
**89**	Serum sum retinyl esters: SI (umol/L)	0.0%	0.0%
**90**	Red cell distribution width (%)	7.1%	4.8%
**91**	Red cell distribution width:SI(fraction)	6.0%	3.6%
**92**	Serum total calcium (mg/dL)	0.0%	0.0%
**93**	Serum total calcium: SI (mmol/L)	0.0%	0.0%
**94**	Serum selenium (ng/mL)	0.0%	1.4%
**95**	Serum selenium: SI (nmol/L)	0.0%	1.4%
**96**	Serum iron (ug/dL)	0.0%	0.0%
**97**	Serum iron: SI (umol/L)	0.0%	0.0%
**98**	Serum glucose (mg/dL)	1.4%	2.7%
**99**	Serum glucose: SI (mmol/L)	1.4%	2.7%
**100**	Serum potassium: SI (mmol/L)	0.0%	0.0%
**101**	Serum total bilirubin (mg/dL)	0.0%	0.0%
**102**	Serum total bilirubin: SI (umol/L)	0.0%	0.0%
**103**	Serum cholesterol (mg/dL)	2.5%	4.9%
**104**	Serum cholesterol: SI (mmol/L)	2.5%	4.9%
**105**	Serum triglycerides (mg/dL)	6.2%	4.9%
**106**	Serum triglycerides: SI (mmol/L)	6.2%	4.9%
**107**	Serum TIBC (ug/dL)	0.0%	0.0%
**108**	Serum TIBC: SI (umol/L)	0.0%	0.0%
**109**	Serum total protein (g/dL)	0.0%	0.0%
**110**	Serum total protein: SI (g/L)	0.0%	0.0%
**111**	Serum triglycerides (mg/dL)	4.1%	2.7%
**112**	Serum triglycerides: SI (mmol/L)	4.1%	2.7%
**113**	Serum uric acid (mg/dL)	1.4%	1.4%
**114**	Serum uric acid: SI (umol/L)	1.4%	1.4%
**115**	Urinary albumin (ug/mL)	1.3%	0.0%
**116**	Urinary cadmium (ng/mL)	2.5%	1.3%
**117**	Urinary cadmium: SI (nmol/L)	2.5%	1.3%
**118**	Urinary iodine (ug/dL)	0.0%	0.0%
**119**	Urinary creatinine (mg/dL)	3.8%	5.1%
**120**	Urinary creatinine: SI (mmol/L)	3.8%	5.1%
**121**	Serum vitamin A (ug/dL)	6.2%	4.9%
**122**	Serum vitamin A: SI (umol/L)	6.2%	4.9%
**123**	Serum vitamin B12 (pg/mL)	0.0%	1.2%
**124**	Serum vitamin B12: SI (pmol/L)	0.0%	1.2%
**125**	Serum vitamin C (mg/dL)	0.0%	0.0%
**126**	Serum vitamin C: SI (mmol/L)	0.0%	0.0%
**127**	Serum vitamin E (ug/dL)	1.2%	3.7%
**128**	Serum vitamin E: SI (umol/L)	1.2%	3.7%
**129**	White blood cell count	2.4%	2.4%
**130**	White blood cell count: SI	2.4%	2.4%

## Discussion

In this era of personalized medicine, research is now focused on identifying specific biomarkers to tailor the therapeutic approach to both the disease and the unique genetic makeup of the patient. This concept is rapidly advancing in oncology, where differences in the genetic composition of a single tumor may be exploited to select individual targeted therapies. Until now, the search for personalized therapeutic strategies has not taken the impact of gender into consideration. Our study emphasizes that gender may be responsible for significant differences in cancer outcome prevalently in patients 17–61 years of age. These differences have been underestimated in previous studies that did not consider the significance of age for the gender effect. To our knowledge, this is the first study that systematically investigates the gender effect stratified over age as continuous variable using US population data. The only other study that has investigated the gender effect with reference to age was conducted in Europe and found a 5% gender-based survival difference [6], as compared to the 30% effect reported here. This discrepancy could be explained by the heterogeneous European database, overrepresentation of older patients or the study design in which age ranges were chosen arbitrarily [6]. Our initial hypothesis was that, if present, the gender effect would be influenced by age, since the hormonal differences between males and females are maximal in the fertile years; similarly, we expected the gender effect to decrease in influence following those years. This hypothesis was confirmed by our analysis since the gender effect peaked during the fertile years, when hormonal differences are maximal by gender.

What determines the gender effect? So far, the concept of hormone-dependent disease has been confined to prostate and breast cancer, where anti-hormone strategies are principal modalities of therapy. Our findings suggest that sex hormones, more generally, could be key drivers of a malignancy's aggressiveness, particularly when cancer is developed at a young age, and may thus be exploited to increase cancer survival rates.

Another important finding in our study is that out of 65 physiologic variables [7], free testosterone displayed the strongest homology to that of the HR. The gender effect has traditionally been explained to result from differences in estrogen levels in the female population, with focus on a female's pre- or post-menopausal status. Our study strongly suggests that also androgens could be involved in driving the gender effect. Indeed, the amount of free testosterone in males is not constant throughout life [8]. Rather, levels increase at around 17 years, peak in the mid-twenties and gradually decrease thereafter until returning to pre-puberty levels at the age of 61 years.

In our study, the gender effect was more prominent in African-American than in Caucasian. In the US population, young African-Americans exhibit higher bone density and muscle mass [9], all parameters which have been related to increased androgen levels [10]. At the same time, there is also an increased risk of prostate cancer in African-Americans which has been correlated with higher levels of androgens [11]. For these reasons, African-Americans may benefit more of a therapeutic manipulation of the hormonal levels aimed at increasing the effects of metastatic cancer treatments. In addition to free testosterone, we noticed that also the amount of hemoglobin displayed a significant correlation with the gender effect in males but not in females. Hemoglobin levels are known to depend on free testosterone levels in males [12], thus strengthening the biological link between HR trend in males and circulating androgen levels.

How are androgens involved in cancer mortality? Here we reported that the gender effect is not visible in all the patients, but only when the disease is solid (epithelial and sarcomas but not hematological malignancies) and at an advanced stage which would require additional treatments. This fact suggests the presence of a relationship between gender effect and response/resistance to treatments used for metastatic cancers. Recently, androgens have been reported to activate a prosurvival pathway in colorectal cancer through the overexpression of class III and V β-tubulin isotypes [13]. Class III β-tubulin is an adaptive survival pathway to a harsh microenvironment featured by hypoxia [14] and poor nutrient supply [15]. In this context, androgens could activate a survival pathway regardless of exposure to such a microenvironment, making a cancer more aggressive and resistant to anoikis, which occurs in the setting of low oxygen and nutrient supply. This would enable cancer cells to metastasize locally and distantly and escape from cancer treatments. These processes could establish a biological ground to explain an androgen-dependent gender effect.

But, do estrogen levels exert some protective effects for cancer survival? This hypothesis, originated by Adami and coll. in 1990 [16], cannot be directly excluded in our study, as the NHANES III dataset did not analyze estrogen levels in its female population. However, other female-specific sex hormones whose expression is directly related to estrogen levels, such as FSH and LH [17], were investigated in the NHANES III population. None of these hormones exhibited a direct relationship with the HR distribution with both Spearman and KS-test. Moreover, estrogen production in females peaks at around 12 years of age [18] and decreases at around 50–51 years of age [19], which is earlier than the pattern of free testosterone in males. Such physiological observations suggest that the curve of estrogen production does not match the HR distribution over age reported here.

The major limitation of our study is that all the results are driven from patient population studies and that SEER database and NHANES III include cancer patients and healthy subjects, respectively. Therefore, our analysis was made from two independent subsets and data did not come from the same patients. However, such risk is partially mitigated by the size of the studied populations and the fact that they were coming across US, thus decreasing the risk to be affected by specific treatments delivered in a single Institution.

Nevertheless, our data emphasize the new hypothesis that androgens, rather than estrogens, could be drivers of the gender effect. An array of antiandrogen therapies has been developed for the management of prostate cancer, including drugs that also decrease tissue production of androgens [20]. Our population study supports the need of prospective clinical trials to test whether young male cancer patients (aged less than 61 years) with metastatic disease could benefit from therapeutic modulation of male hormone levels.

## Supporting Information

Video S1 **Survival analysis from age 0 to 84.** The video is generated by Kaplan-Meier analysis from the data presented in the manuscript from the age 1 to 84 and the animation is obtained with the overlapping of the 84 images. For each age, blue and red lines indicate the survival curve for male and females, respectively.
(MP4)
